# Autoimmunity and hernia mesh: fact or fiction?

**DOI:** 10.1007/s10029-023-02749-4

**Published:** 2023-02-04

**Authors:** B. Jisova, J. Wolesky, Z. Strizova, A. de Beaux, B. East

**Affiliations:** 1 3Rd Department of Surgery, Motol University Hospital, Prague, Czech Republic; 2 Department of Immunology, Motol University Hospital, Prague, Czech Republic; 3 Department of Surgery, Royal Infirmary of Edinburgh, Edinburgh, UK

**Keywords:** Hernia mesh, Immune, Autoimmune, ASIA, Autoimmunity, Polypropylene

## Abstract

**Background:**

There is an increasing number of patients following hernia surgery with implanted mesh reporting symptoms that could indicate autoimmune or allergic reactions to mesh. ‘Allergy’ to metals, various drugs, and chemicals is well recognised. However, hypersensitivity, allergy or autoimmunity caused by surgical mesh has not been proven by a scientific method to date. The aim of this study was twofold: to describe the pathophysiology of autoimmunity and foreign body reaction and to undertake a systematic review of surgical mesh implanted at the time of hernia repair and the subsequent development of autoimmune disease.

**Methods:**

A systematic review using the PRISMA guidelines was undertaken. Pubmed (Medline), Google Scholar and Cochrane databases were searched for all English-written peer-reviewed articles published between 2000 and 2021. The search was performed using the keywords “hernia”, “mesh”, “autoimmunity”, “ASIA”, “immune response”, “autoimmune response”.

**Results:**

Seven papers were included in the final analysis—three systematic reviews, three cohort studies and one case report. Much of the current data regarding the association of hernia mesh and autoimmunity relies on retrospective cohort studies and/or case reports with limited availability of cofounding factor data linked to autoimmune disease such as smoking status or indeed a detailed medical history of patients. Three systematic reviews have discussed this topic, each with a slightly different approach and none of them has identified causality between the use of mesh and the subsequent development of autoimmune disease.

**Conclusion:**

There is little evidence that the use of polypropylene mesh can lead to autoimmunity. A large number of potential triggers of autoimmunity along with the genetic predisposition to autoimmune disease and the commonality of hernia, make a cause and effect difficult to unravel at present. Biomaterials cause foreign body reactions, but a chronic foreign body reaction does not indicate autoimmunity, a common misunderstanding in the literature.

## Introduction

Hernia repair is one of the most common surgical procedures and in most cases involves the use of a surgical mesh. There are many different types of mesh available, the most common material used is polypropylene (PP), a permanent or non-absorbable synthetic polymer. Some surgical meshes are made of other polymers or even composed of more than one material [1, 2].

The use of mesh in hernia surgery significantly decreases hernia recurrence [2, 3], but the permanent nature of the implanted material possesses a lifelong risk of possible complications like infection, foreign body reactions or mesh erosion into neighbouring organs, as seen for example when a polypropylene mesh is in contact with bowel. There are a number of studies investigating the impact of mesh implantation on the acute systemic inflammatory immune response, although this does not indicate autoimmunity [3–6]. Implantation of any foreign body initiates an acute inflammatory reaction which in some cases, progresses to a chronic condition. This implantation leads to a foreign body reaction rather than hypersensitivity or an autoimmune reaction. Yet, it is not clear whether the foreign body reaction can also promote other immunological reactions, such as autoimmune/autoinflammatory reactions, in genetically predisposed patients [1, 3].

The effects of the synthetic foreign body were well described in patients with silicone breast implants [6]. Local complications, such as pain, redness, and swelling are reported along with systemic complications, including arthralgia, fatigue, immunodeficiency, or autoimmune reactions [3, 4, 7, 8]. Some patients do report various immune-related symptoms following the implantation of surgical mesh [4, 7]. The immune responses mediated by implanted biomaterials can be influenced by the type of implanted material, the underlying immune condition or immune status of the person, and many different aspects which vary among individuals, such as smoking history, obesity, medication or physical stress. Furthermore, meshes with larger pores (over 1 mm) tend to have a decreased inflammatory reaction with a lower level of clinical complications [9].

Studies on innate and adaptive immunity in patients after PP mesh implantation show that both components of the immune system are involved in the immune response to a foreign body [10]. Both adaptive and innate immune cells are triggered by the foreign body to produce a large amount of pro-inflammatory cytokines. The binding of pro-inflammatory cytokines to their receptors causes a subsequent activation of a signalling cascade that eventually provokes the activation of immune cells, the expression of cell cycle regulators, pro-apoptotic proteins, and adhesion molecules [11]. This initial inflammatory state may result in autoimmune organ-specific damage [12]. Cytokines with prevailing pro-inflammatory activities, such as IL-6, IL-1, TNFα, IL-18, IL-33, and IL-36, are known to regulate the recruitment and activation of effector immune cells and they are largely involved in the pathogenesis of chronic inflammation [12]. Another critical aspect of chronic inflammation is the process of pathological pain, which has been attributed to the presence of IL-1β, IL-6, and TNF-α cytokines [13, 14]. In general, proinflammatory cytokines directly modulate neuronal activity leading to the development of inflammatory and neuropathic pain [13, 14].

Surgery-associated trauma also generates an inflammatory response, which is further associated with the activation of the complement system, release of coagulation factors, induction of cytokine transcription, and activation of antibody secretion. Markers of the inflammatory response usually normalize within 7 days after the surgery. Nevertheless, prolonged inflammation may cause generalized symptoms or initiate the production of autoantibodies [1, 6, 12–14]. Identification of different autoantibodies can be associated with specific autoimmune diseases. Nevertheless, almost all autoantibodies, including antinuclear antibodies (ANA), are often found in healthy individuals [15]. There is a growing body of evidence that the presence of a low titre of autoantibodies is linked to immune activation and protection against foreign intrusions rather than to the confirmation of an autoimmune disease [15]. Autoantibodies are also commonly found in infectious diseases [16]. Thus, the existence of physiological autoimmunity should not automatically lead to the diagnosis of autoimmune disease because the auto-recognition of a foreign particle corresponds with a normal protective function of the immune system [15].

## Autoimmune/inflammatory syndrome induced by adjuvants

Symptoms associated with the systemic use of biomaterials have been described as the autoimmune inflammatory syndrome induced by adjuvants (ASIA)—Shoenfeld´s syndrome. During the last 50 years, these symptoms were previously addressed also as human adjuvant disease, silicosis, silicone incompatibility syndrome, and most recently, ASIA. ASIA was first described in 2011 by Shoenfeld et al. [9, 17, 18].

A genetic predisposition plays a crucial role in the development of autoimmune diseases in general. The clinical manifestation, however, is largely dependent on the environmental factors that trigger the autoimmune response in genetically predisposed patients. Obesity, smoking and physical stress are presumably the most important environmental factors contributing to the development of autoimmunity [19]. It has been also demonstrated that early life stress has a large impact on subsequent inflammatory responses [20]. However, even though multiple genes contribute to the development of autoimmune disease, *genetic* predisposition always requires environmental *triggers* to clinically manifest [21].

Adjuvants are substances that were shown to induce robust immune responses in both humans and animals. In vaccinology, adjuvants serve as promotors of antigen-specific immune reaction and allow patients to mount significantly higher titres of pathogen-specific antibodies after vaccination [9, 17]. Well-known adjuvants are silica, aluminium hydroxide, and also silicone. It has recently been suggested that PP mesh could also be an adjuvant, but this has never been proven to date.

Immediately after the insertion of surgical mesh, a layer of fibrinogen and other host proteins is formed and deposited onto the biomaterial. Fibrinogen has a specific peptide region responsible for attracting and binding macrophages and other phagocytes [1, 4, 8, 20, 22]. Adjuvant particles are captured by macrophages and further processed in phagosomes and phagolysosomes. This process leads to the activation of inflammasomes and subsequent stimulation of a cascade of kinases which produce a pro-inflammatory signal leading to the activation of NFκB. Pro-inflammatory cytokines, mainly the members of the IL-1 cytokine family, generate a massive inflammatory response. Several pro-inflammatory cytokines, such as IL-1a and IL-33 may also be released as alarmins from damaged cells [23]. Within these processes, reactive oxygen species (ROS) and reactive nitrogen species (RNS) are produced. In addition, apoptosis of macrophages is accompanied by the release of adjuvants-containing particles that again, serve as powerful stimuli to macrophages resulting in either wound healing or acute tissue damage. To understand the balance between immune-mediated wound repair and tissue damage leading to a chronic disease, it is crucial to clarify the role of immune cells in the pathogenesis of tissue injury [24].

Currently, data focusing on the exact pathophysiology of mesh implantation are very limited. So far, it has been shown that exposure to adjuvants-containing particles leads to the production of interleukin-17 (IL-17). IL-17 supports an influx of neutrophils that are activated and produce ROS and release enzymes such as myeloperoxidase. In addition, adjuvants-containing particles are transported to the regional lymph nodes, resulting in a pronounced adjuvant effect and enhancement of the adaptive immune response to an autoantigen [9, 19, 25, 26]. To this date, there is no published study looking into IL-17 production and a hernia mesh. Moreover, studies evaluating the local recruitment of immune cells at the site of mesh implantation, as well as the complex involvement of diverse cytokines and chemokines, are completely lacking. In a study by Patti et al., hernia mesh is shown to trigger higher production of IL-1 in its vicinity but not in tissue distant to it [27].

**Fig. 1:**
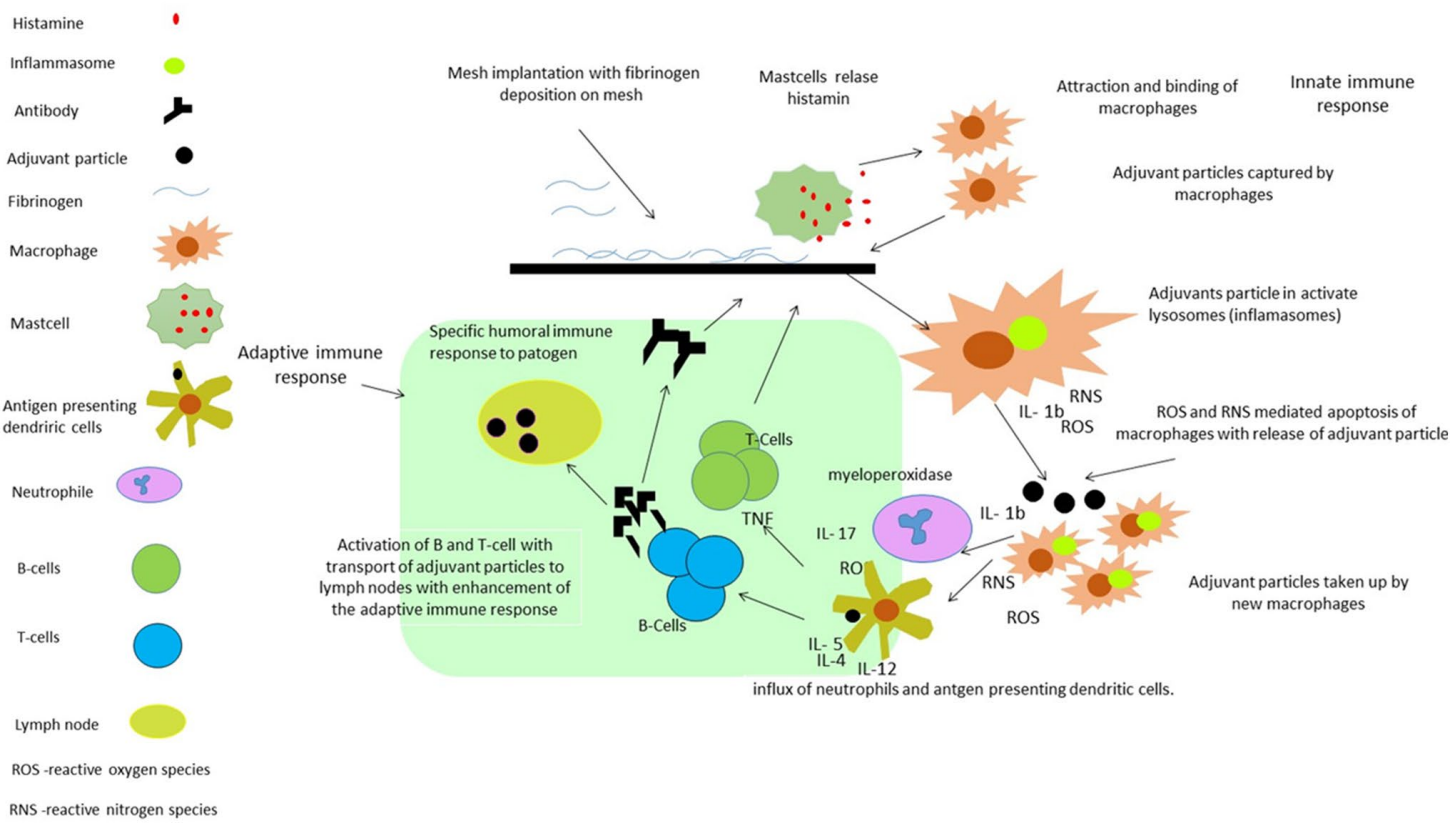
Schematic diagram of possible mechanism of development of auto-immune reaction to implanted synthetic mesh

The patho-physiological process of possible auto-immunity to synthetic mesh is illustrated in Fig. 1.

## Diagnostic criteria of autoimmune/inflammatory syndrome induced by adjuvants

ASIA syndrome has major and minor diagnostic criteria.

Major criteria are:

- Exposure to an external agent (infection, vaccine, or adjuvant) before clinical manifestation
- The appearance of typical clinical manifestations: myalgia, myositis, muscle weakness, arthralgia or arthritis, chronic fatigue, sleep disorders
- Neurological deficiencies associated with demyelination, cognitive impairment and/or memory loss
- Pyrexia
- Removal of the inducing agents causes significant improvement in symptoms.
- Affected organs exhibit typical signs of inflammation on biopsy

Minor criteria are:

- Identification of autoantibodies or adjuvant-specific antibodies
- Other clinical manifestations (such as irritable bowel syndrome]
- Specific HLA association (HLA-DRB1 or HLA-DQB1)
- Initiation/exacerbation of an autoimmune disease (multiple sclerosis or systemic sclerosis) [3, 7, 9, 17].

While in many autoimmune diseases, such as rheumatoid arthritis (RA) or systemic lupus erythematosus (SLE), diagnostic criteria are based on specific laboratory findings in association with clinical signs, in ASIA syndrome, we lack such specific criteria, with the potential to over-diagnose the syndrome. Generally, the serum C-reactive Protein level is normal in ASIA, and angiotensin-converting enzyme (ATCE) together with interleukin 2 (IL-2) are elevated. However, a wide variety of factors, such as ageing or smoking, can affect the serum levels of IL-2 and other pro-inflammatory cytokines. [28].

A quarter of the patients who develop ASIA were found to have elevated IgG levels and/or specific IgG subclass levels and vitamin D deficiency, but again, both these laboratory variables may accompany a variety of health conditions and thus, *the results of these studies should be interpreted carefully* [4, 19]. The elevations in these IgG levels are in no way pathognomic of ASIA.

## Methods

The PRISMA [27] guidelines were followed in this systematic review. Two researchers performed the initial search and then screened by name and abstract of the article. All researchers have performed the final screening of the full texts and data extraction.

**Fig. 2:**
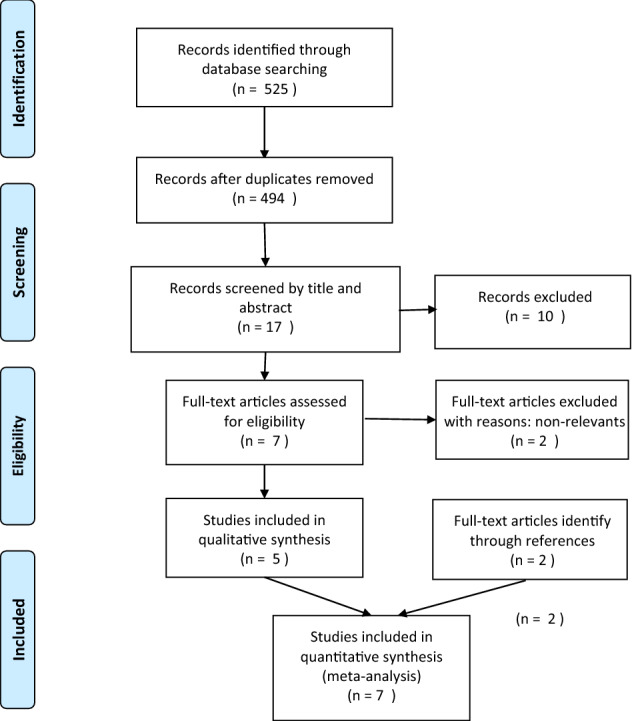
Prisma flow chart of literature search

Pubmed (Medline), Google Scholar, and Cochrane were searched for all English-written and peer-reviewed reports published in indexed international journals until January 2022. The keywords used were “hernia”, “mesh”, “autoimmunity”, “ASIA”, “immune response”, “autoimmune response”. The PRISMA flow chart is shown in Fig. 2. The SIGN checklist was used to assess the quality of all selected articles.

## Results

Seven articles comprising 3 systematic reviews, 3 cohort studies and 1 case report were included as presented in Table 1. All of the available articles were rated as low-quality scientific evidence with a high risk of bias. As the three systematic reviews included were also rated of low quality, we have analysed the original sources rather than the SR themselves.

**Table 1:** Summary of papers included in the review

First author	Year	Title of study	Aim of study	Study type	Patients and material	Methods	Outcomes
Clancy [1]	2019	Polypropylene mesh and systemic side effects in inguinal hernia repair: current evidence	To evaluate the systemic reaction to hernia mesh implantation	Systematic review	23 articles on polypropylene mesh and systemic reaction	Medline, National Library of Medicine, Embase and Cochrane Library	No evidence to link polypropylene mesh and systemic or auto-immune responses
Dias [3]	2012	Autoimmune [auto-inflammatory] syndrome induced by adjuvants (ASIA): Case report after inguinal hernia repair with mesh	Case report of ASIA after hernia mesh implantation	Case report	Patient with silicon breast implants and bilateral inguinal mesh hernioplasty	Clinical examination, mesh explanation and histology	Mesh removal in the case improved systemic symptoms
Teravaer [4]	2018	Autoinflammatory/autoimmunity syndrome induced by adjuvants (Shoenfeld’s syndrome) in patients after a polypropylene mesh implantation	Identify patients with ASIA after polypropylene mesh implantation and investigate changes in immune response	Prospective cohort	714 patients, 40 had symptoms of ASIA. 18 mesh hernioplasty and 22 vaginal mesh	Clinical, histology and laboratory investigations	Fatigue common symptoms of ASIA. Pre-existing allergic conditions a risk factor for ASIA. ASIA is associated with lower serum IgG
Kowalik [29]	2020	Are polypropylene mesh implants associated with systemic autoimmune inflammatory syndromes? A systematic review	Looking for correlation between polypropylene mesh implantation and autoimmune reaction	Systematic review	4 papers on topic	Medline, Embase, Web of Science, Scopus and Cochrane Library	No association between polypropylene mesh implantation and systemic autoimmune syndrome
Kotovic [30]	2017	Systemic inflammatory response after hernia repair: a systematic review	An overview of inflammatory response and serum markers following hernia repair – comparison of markers between mesh and non-mesh repairs	Systematic review	31 papers on systemic inflammatory response after hernia mesh repair	Medline, Embase and Scopus	Hernioplasty increased serum CRP, IL-6, leukocyte, neutrophil, IL-1, IL-10, fibrinogen and alpha1-antitrypsin, with a decrease in lymphocyte count and albumin at 24 h. Slightly higher responses in those with a mesh implant
Dievernich [10]	2012	Characterization of innate and adaptive immune cells involved in the foreign body reaction to polypropylene meshes in the human abdomen	Investigation of immune response, especially adaptive immune response, to foreign body implants in the human abdomen	Retrospective cohort	7 patients with polypropylene mesh ventral hernia repair	Histology with immunofluorescence analysis	Demonstrated both innate and adaptive immunity contributes to a foreign body reaction after mesh implantation
Chughtai [43]	2017	Hernia repair with polypropylene mesh is not associated with an increased risk of autoimmune disease in adult men	Comparison of patients with mesh implantation to control cohort (patients undergoing colonoscopy) and looking for development of ASIA in either group	Retrospective cohort	26,579 patients after mesh hernioplasty and 271 after colonoscopy	Patient database analysis	No association between polypropylene mesh implantation and autoimmune disease or ASIA

In brief, the systematic review and meta-analysis by Kowalik et al. included any patient with a PP implant and was not specific to hernia surgery. No causality between the use of PP mesh and autoimmunity was observed [29]. A systematic review by Clancy et al. also did not identify evidence linking PP with systemic autoimmune disease [1]. Indeed, this paper focused mainly on explaining autoimmunity and foreign body reaction in general. Kokotovic et al. published a systematic review looking into the patterns of acute immune reaction after hernia repair, with and without mesh [30]. Hernia surgery was shown to increase CRP, IL-6 levels, leukocyte count, neutrophil count, IL-1 levels, IL-10 levels, fibrinogen, and α1-antitrypsin, and decrease lymphocyte counts and albumin during the first 24 postoperative hours. These inflammatory markers were higher in patients after the mesh implantation, but also in open compared to laparoscopic surgery. However, autoimmunity as such was not discussed.

Similar results were published in a clinical trial by Di Vita et al. In this study, markers of systemic inflammation (IL-6, CRP, alpha-1 antitrypsin, leukocytes and neutrophils) were measured within 7 postoperative days following Lichtenstein and Bassini hernia repair in local anaesthesia. While the authors measured a bigger increase in the early postoperative period, the differences diminished completely within 168 h. Autoimmunity as such was not discussed [31].

Cohen Tervaert et al. found 40 patients with PP mesh implants in a series of 714 patients he examined in his clinic for suspected autoimmunity [4]. 18 of them had a hernia repaired with mesh and most claimed their symptoms started shortly (within 3 years) after the mesh was implanted. 75% of those had a pre-existing allergic disease and two have committed suicide during the follow-up. This paper is a retrospective analysis of a single specialist reporting on 18 people with very little information provided on patient demographics including important ones such as smoking status and obesity. Only 2.5% of the patients in this clinic had hernia mesh implanted and 5.6% had any PP implant, which is likely less than the general population given that hernias and hernia repair are such a common operations.

Chuguthai et al. provided a retrospective comparative analysis of 26 575 patients after hernia mesh repair and 71 271 healthy controls who underwent a colonoscopy [10]. 1.6% of patients in the mesh group and 1.7% of patients in the control group had developed one of the recognised autoimmune disorders during the follow-up period (2 years). At six months, the risk was 0.1% vs 0.1%, at one year 0.2% vs 0.2% and at 2 years 0.3% vs. 0.3%. The authors concluded that a polypropylene mesh can undergo oxidative degradation and cause chronic inflammation and pain but there was no clear association between the mesh implantation and the development of autoimmune disease. However, no histological examination is included in the study and despite people being matched for comorbidities, race and age during the analysis of the data, smoking status was not known.

A case report published by Dias et al. reported on a 33 years old woman after silicone breast implantations and bilateral groin hernioplasty and implied that there was a link between implanted hernia mesh and the patients’ systemic symptoms [3]. The patient suffered from bilateral chronic groin pain and both of the meshes were explanted after which not only the pain but also the systemic symptoms diminished. Histological examination showed inflammatory lymphocyte infiltration and chronic granuloma with giant cells, which was considered proof of an autoimmune reaction by the authors. However, we emphasize that these histological findings refer more likely to be a chronic foreign body reaction rather than autoimmunity. It is a single case report, the patient was a smoker and was left with breast silicone implants, yet the systemic symptoms disappeared. It is surprising given the fact than silicon has been repeatedly linked to autoimmunity including laboratory signs while this is not the case for polypropylene mesh [32].

## Discussion

This study has demonstrated the paucity of evidence to link hernia mesh, in particular polypropylene mesh, to the development of subsequent autoimmune disease. As all autoimmune diseases have a genetic predisposition, the critical point of most of the presented studies is the lack of genetic testing in the selected individuals. Furthermore, the inflammatory response to foreign materials belongs to physiological immune responses that should be studied with respect to all the immune cells, cytokines and chemokines present at the site of inflammation. The clinical evidence is retrospective in nature and contains a high risk of bias including heterogeneity of patients, different operations, and assessment methods, and most importantly, data regarding the patients’ comorbidities and medical history were not taken into consideration.

As environmental factors play a pivotal role in triggering autoimmune diseases, considering mesh implantation a single cause of autoimmunity is highly misleading. In the studies reviewed, no information was given as to what, if any, patient screening for autoimmunity prior to their hernia operation was undertaken, or what autoantibodies they were tested for—all leading to these studies having a high risk of bias. The lack of sensitivity or specificity of the majority of current inflammatory markers and autoantibodies measured, including their presence in healthy people, makes extrapolation of these to confirm the presence of a specific autoimmunity to hernia mesh scientifically unsound. The single comparative study comparing people with and without hernia mesh [10], showed a similar diagnosis rate of autoimmune disease. While this does not discount the possibility of hernia mesh-induced autoimmune disease, it would imply that such a possibility is a rare event. We thus disagree with Kowalik et al. who reported that the same included studies had a low risk of bias [20].

It is not always scientifically sound to extrapolate the findings between various types of materials (silicon, infectious agents, aluminium salt, vaccines, and other foreign material). Of the implantable materials, the one most studied is silicone because silicone implants have been used since the 1960s [33]. The first autoimmune syndrome after mammoplasty was described in Japan in 1964. It was named “Human adjuvant disease” [34]. In the 1980s many more case reports and series of patients with some autoimmune disease after silicone breast implants were published. Over the last decade, a deeper understanding of implanted foreign materials in humans and their potential link to autoimmunity has been gained [35]. It is mentioned that there is a significant underdiagnosing of ASIA syndrome due to the lack of knowledge among diagnosing clinicians and the list of possible causative agents is slowly growing in cosmetic surgery. However, there is not a growing body of evidence supporting polypropylene being among those adjuvants [36].

The use of synthetic mesh in hernia repair is well-established [1, 2]. Indeed, hernia guidelines recommend mesh in most hernias to minimise hernia recurrence. Local complications are well described clinically yet they are not quite so well understood from the histological perspective [10]. For example, it is known that approximately 10–20% of patients after inguinal hernia repair suffer from chronic pain to some degree [5, 35]. However, causes of this pain are variable and most likely linked to nerve injury rather than to mesh-related autoimmunity. To add to this confusion, many authors incorrectly mix autoimmunity and chronic foreign body reaction. Yet, this distinction is important as they are different pathophysiological processes. The processes of chronic foreign reaction have been described in both experimental and human settings and have never suggested an autoimmunity type of reaction. [36]. In fact, it was observed, that the blood levels of various cytokines including IL-1 decrease to preoperational levels within days after the operation regardless of the type of polypropylene material used. [37].

Another factor that may influence the response of the host to the mesh implant is the anatomical position of mesh placement. While pelvic mesh is at times in direct contact with viscera and can cause a plethora of symptoms, a mesh hidden or buried within the layers of the abdominal wall is perhaps unlikely to cause for example irritable bowel syndrome. The type of collagen produced differs based on the position of the mesh within the abdominal wall. [38].

So, it is likely that the type of immune reactions associated with hernia mesh use might vary depending on the mesh position and perhaps what organs that the material comes in contact with.

Autoimmune disease is common with increasing age, as is hernia disease. It is estimated that between 4 and 8% of the population is affected by one of 80 known autoimmune diseases. [37] There are many medical and psychological conditions that can lead to autoimmunity in genetically predisposed people. It is likely that even the operation trauma itself can trigger autoimmunity in some people, but the presence of a permanent implant is an easy thing to blame. The lack of good research in this area has added to the belief of many that the use of mesh has led to their autoimmune disease. And thus, the whole “mesh injured” phenomena grows. Sadly, patients with autoimmunity are more likely to suffer from mental health issues and more prone to self-harm, adding to this emotive topic [39]. Nevertheless, with more than twenty million meshes being implanted annually worldwide, it is somewhat surprising that there are not more reports linking hernia mesh to various auto-immune disorders. Whether the operation itself [40] or the use of any implantable material or indeed another factor such as a viral infection is the cause of autoimmune diseases related to hernia mesh requires further study. Susceptibility to such triggers is very variable in the population. PubMed lists more than 12,000 articles dedicated to potential triggers of autoimmunity [40, 41].

What next for the hernia community in this area of research? To evaluate the immunogenicity of surgical mesh, the cellular immune response should be assessed as the proportion of reactive CD4 + and CD8 + T cells producing pro-inflammatory cytokines after ex vivo stimulation with polypropylene mesh and co-stimulation with costimulatory antibodies [42].

We also need more follow-up data asking specific questions around symptoms of autoimmune disease. This needs to be a joint effort between hernia surgeons and other healthcare professionals in autoimmune disease. This may allow the identification of a group of people who could help us confirm a causal link if such a link does exist. And if this link exists, it helps develop reliable clinical laboratory tests to make the diagnosis in those affected quick, easy and reliable. And then research strategies to aid the clinical management of these patients, as to what to do with the hernia mesh if such a condition develops.

## Conclusion

Currently, there is little evidence that the use of polypropylene mesh can lead to autoimmunity. A large number of potential triggers of autoimmunity along with the genetic predisposition to autoimmune disease and the commonality of hernia, make a cause and effect difficult to unravel at present. Biomaterials cause foreign body reactions, but a chronic foreign body reaction does not indicate autoimmunity, a common misunderstanding in the literature.
